# Identifying and characterizing high-risk populations in pilot malaria elimination districts in Madagascar: a mixed-methods study

**DOI:** 10.1186/s12936-024-04927-w

**Published:** 2024-04-26

**Authors:** Elisabeth Gebreegziabher, Andry Raoliarison, Andrinirina Ramananjato, Andriamamonjy Fanomezana, Martin Rafaliarisoa, Sandy Ralisata, Jocelyn Razafindrakoto, Jennifer L. Smith, Jehan Ahmed, Cara Smith Gueye

**Affiliations:** 1 Malaria Elimination Initiative, University of California San Francisco, San Francisco, CA USA; 2 US President’s Malaria Initiative (PMI), PMI Impact Malaria, Antananarivo, Madagascar; 3 National Malaria Control Programme, Antananarivo, Madagascar; 4 U.S. President’s Malaria Initiative, USAID, Antananarivo, Madagascar; 5 U.S. President’s Malaria Initiative (PMI), PMI Impact Malaria, Washington, DC USA; 6 Department of Epidemiology and Biostatistics, University of California, San Francisco, CA USA

**Keywords:** Malaria, Malaria elimination, High risk populations, Hard to reach populations, Vector control, Surveillance, Madagascar, Subnational tailoring, Targeting

## Abstract

**Background:**

In Madagascar, the districts of Antsirabe II, Faratsiho and Antsiranana I have relatively low malaria incidence rates and have been selected by the National Malaria Control Programme for pilot elimination strategies. The districts have residual transmission despite increasing coverage and quality of malaria services. This study sought to identify priority subpopulations at highest risk for malaria and collect information on intervention preferences and methods that will inform subnational tailoring of malaria service delivery.

**Methods:**

This mixed methods study employed (i) a quantitative malaria risk factor assessment in Antsirabe II and Faratsiho comprising a test-negative frequency matched case–control study and a qualitative risk factor assessment in Antsiranana I; and (ii) a qualitative formative assessment in all three districts. For the case–control study, a mixed effects logistic regression was used with age, sex and district included as fixed effects and health facility included as a random effect. The qualitative risk factor assessment used semi-structured interview guides and key informant interviews. For the qualitative formative assessment in the three districts, a summary report was generated following semi-structured interviews and focus group discussions with high-risk populations (HRPs) and stakeholders.

**Results:**

In Antsirabe II and Faratsiho districts, rice agriculture workers, outdoor/manual workers, particularly miners, and those with jobs that required travel or overnight stays, especially itinerant vendors, had higher odds of malaria infection compared to other (non-rice) agricultural workers. In Antsiranana I, respondents identified non-rice farmers, mobile vendors, and students as HRPs. Risk factors among these groups included overnight stays and travel patterns combined with a lack of malaria prevention tools. HRPs reported treatment cost and distance to the health facility as barriers to care and expressed interest in presumptive treatment and involvement of gatekeepers or people who have influence over intervention access or participation.

**Conclusions:**

The study results illustrate the value of in-depth assessments of risk behaviours, access to services and prevention tools, surveillance and prevention strategies, and the involvement of gatekeepers in shaping subnational tailoring to reach previously unreached populations and address residual transmission in elimination settings.

**Supplementary Information:**

The online version contains supplementary material available at 10.1186/s12936-024-04927-w.

## Background

Despite major investments and implementation of interventions across high and low burden countries, progress against malaria has stalled. One explanation for this pattern is that “last-mile” challenges are not affected by current control strategies. These challenges include having difficult to reach populations, outdoor biting vectors, and poor quality health services that all create gaps in malaria care and prevention [1–4]. Tailoring of malaria service delivery for these heterogeneous communities must be done at a subnational level to have sufficient understanding of the populations at risk and the services that would be effective and accepted.

**Fig. 1:**
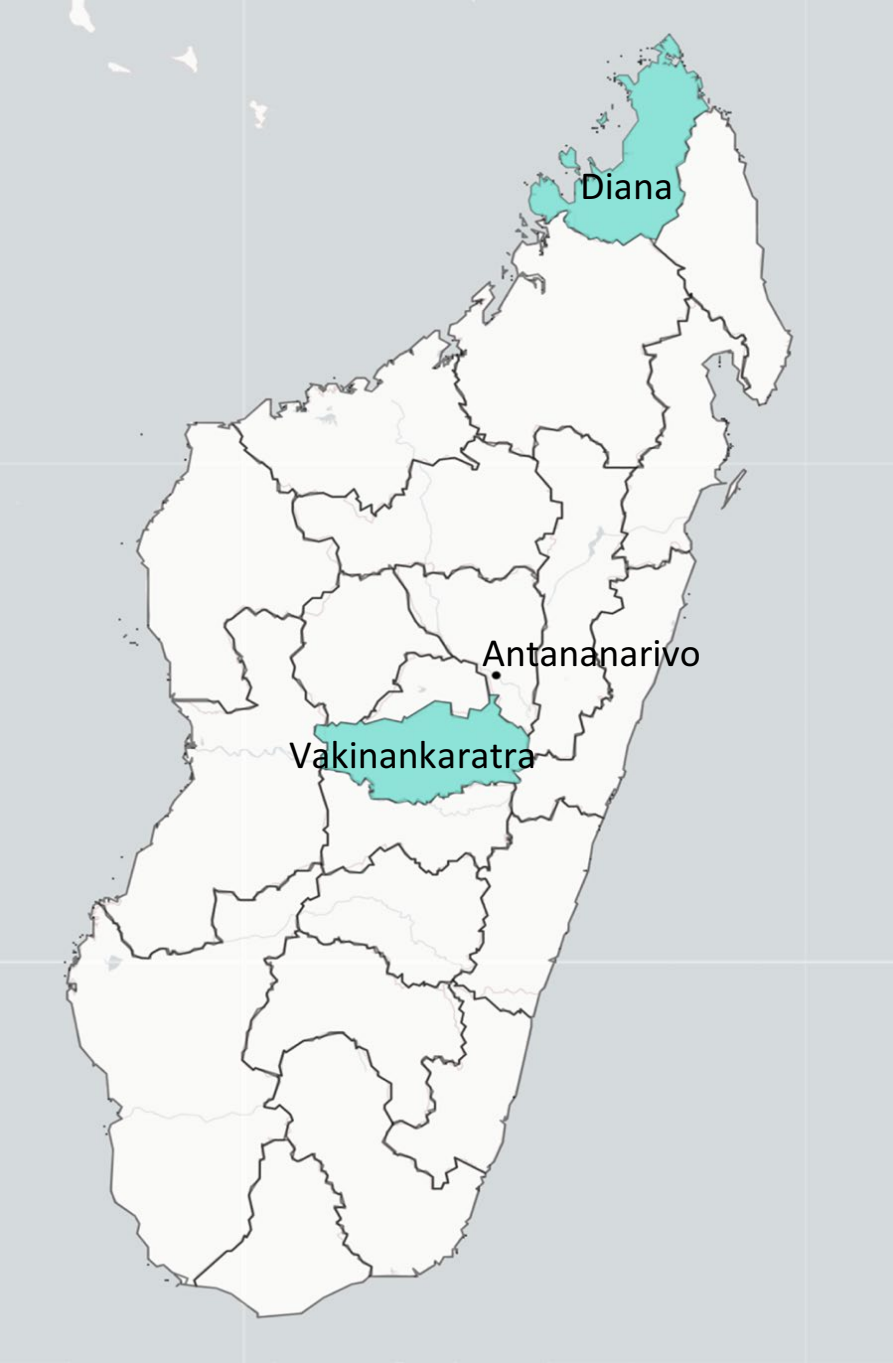
Map of regions included in the project, Vakinankaratra Region (Antsirabe II and Faratsiho districts), Diana Region (Antsiranana I district)

The malaria burden is heterogeneous across Madagascar, with low-transmission areas concentrated in the central highlands and the northern part of the country [5]. The National Malaria Control Programme (NMCP) of Madagascar has increasingly recognized that assessment of and subnational tailoring for local malaria needs is essential for controlling malaria transmission [5]. In 2019, thirteen districts in low-transmission areas were targeted for malaria elimination. Three of these, Antsirabe II and Faratsiho (Vakinankaratra Region) and Antsiranana I (Diana Region) were selected by the NMCP for pilot elimination activities with support from the U.S. President’s Malaria Initiative (PMI) Impact Malaria project (Fig. 1). These districts were selected because they have lower malaria incidence (e.g., API close to less than 1 per 1000) than other districts, but have persistent residual transmission, and they represent rural (Antsirabe II and Faratsiho) and urban (Antsiranana I) areas. However, there is limited information regarding local risk factors for malaria in these regions.

To tailor malaria interventions, districts and national programs need to understand the relative burden of malaria across population groups and important malaria risk factors. Identifying and characterizing groups with persistent transmission and approaches for provision of diagnosis, treatment, and surveillance will help improve malaria responses, such as foci identification, case-based reporting, and case classification. Furthermore, routine vector control may be less effective in remote areas and among highly mobile populations [6]. Therefore, there is also a need for subnational targeting of vector control interventions to groups that may be more difficult to access and may have different behaviours and preferences.

Studies have shown the utility of mixed methods approaches for generating evidence and interpreting data toward intervention design or malaria programmes [7–10]. The University of California, San Francisco’s Malaria Elimination Initiative developed the *Malaria Elimination Guide to Targeted Surveillance and Response in High-Risk Populations* (HRP Guide) to provide a framework for implementing assessments within the local context of a malaria programme to identify high-risk populations (HRPs) and their associated characteristics and risk factors, and use that information to tailor and target interventions [11].

In this mixed methods study, the HRP Guide was used to conduct quantitative and qualitative assessments to identify the most important malaria risk factors, as well as a qualitative formative assessment to collect information on HRP characteristics, behaviours, and preferences that can be used to design interventions for these groups. The overall aims of this project align with the NMCP’s goal to increase access to diagnosis and treatment of malaria, and delivery of health education. This work will provide information to the NMCP for subnational tailoring of malaria services.

## Methods

This mixed-methods study included (i) a malaria risk factor assessment comprising a case–control study in Antsirabe II and Faratsiho and, due to projected low case numbers and lack of power (see below) a qualitative risk factor assessment in Antsinanana I and (ii) a qualitative formative assessment in Antsirabe II, Faratsiho, and Antsiranana I. The methods and objectives by district are presented in Table 1.

**Table 1:** Methods and objectives by district

	Risk factor assessment	Formative assessment
Antsirabe II	Case–control study to identify risk factors linked with highest magnitude of malaria risk	To collect information on HRP characteristics, behaviours, and intervention preferences and potential locations
Faratsiho	Case–control study to identify risk factors linked with highest magnitude of malaria risk	To collect information on HRP characteristics, behaviours, and intervention preferences and potential locations
Antsiranana	Qualitative-based assessment to identify perception of risk groups and risk behaviours	To collect information on HRP characteristics, behaviours, and intervention preferences and potential locations

## Study setting

The Antsirabe II and Faratsiho districts of the Vakinankaratra Region are located in the central highlands and are considered predominantly rural and agricultural, while Antsiranana I district of the Diana Region is located in the north of Madagascar and is considered urban [12]. Malaria transmission in these regions is highest between October and April as it tends to be rainy and hot [13]. *Plasmodium falciparum* is the dominant species causing malaria cases in Madagascar [13, 14]. *Anopheles gambiae *sensu lato (*s.l*.) is considered the primary vector and has shown both indoor and outdoor biting behaviour [15–17]. The malaria epidemiology in the study districts is presented in Table 2.

**Table 2:** Demographics, malaria epidemiology and health facilities in the study districts

	Population (2020)	Confirmed malaria cases (2020)	API^a^ (2020)	Number public health facilities	Number private health facilities
Antsirabe II	475,556	244	0.5	34	8
Faratsiho	237,162	164	0.6	20	2
Antsiranana I	134,922	74	0.5	3	12

^a^Annual Parasite Index, cases per 1000 population

## Risk factor assessment

### Study design and population

The quantitative risk factor assessment in Antsirabe II and Faratsiho was a health facility-based prospective case–control study that used a ‘test-negative’ design to compare the characteristics of individuals with malaria (cases) to those without malaria (controls) within the existing surveillance system. The study was conducted in 18 health facilities in Antsirabe II and 9 health facilities in Faratsiho that had the highest burden of malaria in 2020. In Antsirabe II, in the selected 18 health facilities (of 42 total), there were 221 confirmed cases in 2020, or 90.6% of the total district cases (244). Health facility confirmed cases ranged from 2 to 56 over the 12 month period in 2020, with a mean of 12.28 and a median of 6.5 cases. In Faratsiho, in the 9 selected health facilities (of 23 total), there were 151 confirmed cases in 2020, or 92.1% of the total district cases in 2020 (164). Health facility confirmed cases ranged from 4 to 75 over a 12 month period in 2020, with a mean of 16.78 and a median of 9 cases. The study population included patients routinely tested for malaria at the sampled health facilities. Patients were eligible for inclusion if they attended a selected health facility with suspected malaria, tested positive by rapid diagnostic test (RDT) (if case) or tested negative by RDT (if control), were available to participate in the questionnaire, and provided informed consent. Patients with severe malaria, a prior malaria diagnosis in the preceding month, or a history of malaria prophylaxis or treatment in the preceding 14 days were excluded.

Recruitment targets were determined based on sample size calculations. Assuming a 38% prevalence of exposure in cases and a 20% prevalence in controls, with 80% power and alpha = 0.05, a minimum sample size of 71 cases and 143 controls per district was required to detect an odds ratio of 2.5 with a 1:2 case-to-control ratio. Initial recruitment targets were based on this calculation, but due to the low case burden during the first three months of data collection, the target number of controls was increased in January 2022 to four times that of cases to maximize power. Controls were frequency matched to cases on age, gender, and health facility in a 1:4 ratio. Using a prospective case–control approach, cases and controls were enrolled within the specified distribution of matching factors separately as they visited the study facilities.

The risk factor assessment in Antsiranana I took the form of a qualitative design using a semi-structured interview guide and key informant interviews (KIIs). These methods were used because malaria surveillance data from 2019 to 2020 showed that case numbers would be too low to power a case–control study. The qualitative study was carried out with Chief Medical Officers, district malaria officers, community health workers and leaders in 11 health facility catchment areas with a high burden of malaria.

## Data collection

In Antsirabe II and Faratsiho, data collection was carried out during the peak malaria transmission period from October 2021 to March 2022 (Additional file 1: Fig. 1). A standard questionnaire developed for this assessment was administered by health facility staff using the District Health Information System (DHIS2-University of Oslo, 2021) electronic data collection platform with tablets. Data was collected on participant characteristics and risk factors and included age, gender, primary and secondary occupation, agricultural or field activities at night, overnight travel, intervention coverage, and access to malaria prevention. The questionnaire was translated from English into French and Malagasy, and pilot-tested and revised by the team prior to data collection and with health facility workers during training.

For the risk assessment in Antsiranana I, a semi-structured interview guide for key informant interviews was developed based on the questions used in the case–control study. The questionnaire was translated from English into French and Malagasy, and pilot-tested during the first interview.

## Data management

For the quantitative assessment, detailed information on primary and secondary occupation were categorized for analysis based on theoretical malaria exposure and the number of participants in each occupation. The final occupation categories were (1) “none” and unemployed, (2) agriculture (other than rice), (3) rice agriculture, (4) indoor work (e.g., nurse, teacher, “professional”, student, factory worker, sales or commerce-small market), (5) raising animals or fishing, (6) outdoor work or manual labour (e.g., tree cutter, miner, rock cutter, quarry worker, construction worker), and (7) jobs with travel or overnight stays away from home (e.g., itinerant vendor, driver of truck or passenger vehicle). Since non-rice agriculture had the largest number of participants, it was used as a reference group to compare malaria among all occupation groups.

## Data analysis

The Antsirabe II and Faratsiho data were used to compare risk factors between cases and controls using mixed effects logistic regression, with age (< 14, 14–40, and > 40), sex, and district included as fixed effects and the health facility included as a random effect with a random intercept. Similar mixed models that adjusted for the matching variables (age and sex) were used for analysis of each risk factor/predictor (e.g., occupation, activities, travel, and access to malaria prevention). Estimates across both districts were expressed as odds ratios with 95% confidence intervals. STATA version 14.2 (Stata Corp, Tx) was used for analysis.

Qualitative data from Antsiranana I was recorded in Microsoft Excel during the interviews. The most prevalent responses regarding age, gender, profession, high-risk activities, travel patterns, probability of importing malaria, and locations and times when these groups may be found were assessed for both confirmed cases and for population groups that the respondent believed may be vulnerable to malaria infection but typically do not present at health facilities, referred to in the study as potential malaria cases. If more than one key informant perceived that a certain group was most at risk, that was considered a more prevalent response.

## Formative assessment

### Study design and population

The formative assessment was conducted from June to July 2022 and employed qualitative methods comprising (i) KIIs with HRP group members in Antsirabe II, Antsiranana I, and Faratsiho in the case where focus group discussions (FGDs) were not feasible, such as when a minimum of six group members could not be found, and (ii) FGDs with HRP group members in the three districts. At the end of each KII and FGD, a list of venues was developed with places and times when HRP groups were likely to gather.

Eligible KII and FGD participants in the Antsirabe II and Faratsiho districts were determined based on findings from the case–control study and included individuals aged 18 years of age or older whose primary occupation was rice agriculture worker, itinerant vendor, or miner. In the Antsiranana I district, eligible participants were identified based on the qualitative assessment and included individuals aged 18 years of age and over whose primary occupation was agriculture worker, fisherman, travelling vendor, or student. The questions spanned five themes including risk factors for malaria, malaria HRPs and activities, use of malaria prevention, access to healthcare, operational considerations for access, and acceptability of malaria interventions.

Potential recruitment strategies for surveillance or intervention distribution included peer-referral or venue-based approaches. Peer-referral strategies access populations through recruited individuals referring contacts who meet specified characteristics (e.g., those in the same occupation) [9]. Venue-based recruitment is a technique to access and screen for HRP group members at locations (e.g., shops, markets) where they gather.

### Data collection and management

The formative assessment in the three districts aimed to interview a total of 42 participants, including 36 HRP group members, and to conduct two FGDs with each HRP group. KIIs, FGDs, and venue listings were conducted by a two-person research team of a district health official and a chief medical officer. Instead of transcription, translation and coding of the qualitative data, the study employed a rapid analytic debriefing method whereby the PMI Impact Malaria team conducted a formal debriefing session immediately after each interview and FGD with the moderator and note taker, using a predefined summary template to extract the data most critical to the design of malaria programming [11]. The template was completed in Malagasy then later translated into French and English. The moderator and note taker referred to their notes during this process to ensure accuracy.

### Analysis

A summary report of findings was generated from the thematic analysis of patterns and trends described for each HRP group in the summary debriefings.

**Ethical considerations.**

The Research Ethics Board of Population Services International and the NMCP of Madagascar granted approval of the study. The UCSF Institutional Review Board approved a certification of non-human subjects research due to employees’ lack of access to identifiable data.

## Results

### Risk factor assessment

#### Antsirabe II and Faratsiho

A total of 77 cases and 223 controls were enrolled in Antsirabe II and 45 cases and 88 controls in Faratsiho (Table 3). The cases reported in Faratsiho were below the expected sample size for reasons unknown to the study. In both districts, most of the cases (73.0%) and controls (63.7%) were aged 14–40 years. The majority (59.8%) of participants were male. The most common primary occupation was non-rice agriculture (43.4%) followed by rice agriculture (25.6%). In both districts, jobs that required travel, particularly itinerant vendors, and outdoor workers, particularly miners, were more common among cases than controls (P < 0.05). Over 90% of both the cases and controls resided in the study districts. Most participants did not have access to any type of malaria prevention (such as bed nets, mosquito repellents, or house sprayed with insecticide) (78.1%), did not sleep under an LLIN the previous night (80.6%), did not spend the night outside or travel (87.8%) and did not have outside activities (92.6%) in the previous 3 weeks.

**Table 3:** Characteristics of cases and controls in Antisirabe II and Faratsiho by District

	Antsirabe II						Faratsiho							
	Case (*n* = 77)		Control (*n* = 223)		Antsirabe II total (*n* = 300)		Case (*n* = 45)		Control (*n* = 88)		Faratsiho total (*n* = 133)		Grand total (*n* = 433)	
Age	n	%	n	%	n	%	n	%	n	%	n	%	n	%
14–40	55	71.4%	139	62.3%	194	64.7%	34	75.6%	59	67.0%	93	69.9%	287	66.3%
40 and above	11	14.3%	77	34.5%	88	29.3%	1	2.2%	29	33.0%	30	22.6%	118	27.3%
Less than or equal to 14	11	14.3%	7	3.1%	18	6.0%	10	22.2%	0	0.0%	10	7.5%	28	6.5%
Gender														
Female	20	26.0%	120	53.8%	140	46.7%	5	11.1%	29	33.0%	34	25.6%	174	40.2%
Male	57	74.0%	103	46.2%	160	53.3%	40	88.9%	59	67.0%	99	74.4%	259	59.8%
Primary occupation														
Agriculture (other than rice)	19	24.7%	143	64.1%	162	54.0%	1	2.2%	25	28.4%	26	19.5%	188	43.4%
Farm animal/fishing	4	5.2%	12	5.4%	16	5.3%	0	0.0%	5	5.7%	5	3.8%	21	4.8%
Indoor work	8	10.4%	25	11.2%	33	11.0%	0	0.0%	6	6.8%	6	4.5%	39	9.0%
Jobs with travel/overnight stay away from home	16	20.8%	4	1.8%	20	6.7%	6	13.3%	4	4.5%	10	7.5%	30	6.9%
None	1	1.3%	8	3.6%	9	3.0%	1	2.2%	2	2.3%	3	2.3%	12	2.8%
Outdoor work/ manual labour	7	9.1%	2	0.9%	9	3.0%	5	11.1%		0.0%	5	3.8%	14	3.2%
Rice agriculture	13	16.9%	29	13.0%	42	14.0%	23	51.1%	46	52.3%	69	51.9%	111	25.6%
Reside in the study district														
No	7	9.1%	7	3.1%	14	4.7%	3	6.7%	7	8.0%	10	7.5%	24	5.5%
Yes	70	90.9%	216	96.9%	286	95.3%	42	93.3%	81	92.0%	123	92.5%	409	94.5%
Have access to malaria prevention at home														
No	69	89.6%	184	82.5%	253	84.3%	36	80.0%	49	55.7%	85	63.9%	338	78.1%
Yes-LLIN	8	10.4%	30	13.5%	38	12.7%	9	20.0%	36	40.9%	45	33.8%	83	19.2%
Yes-Other	0	0.0%	5	2.2%	5	1.7%	0	0.0%	0	0.0%	0	0.0%	5	1.2%
Yes-sleep in a tent with LLIN	0	0.0%	4	1.8%	4	1.3%	0	0.0%	3	3.4%	3	2.3%	7	1.6%
Slept under LLIN														
No	68	88.3%	189	84.8%	257	85.7%	39	86.7%	53	60.2%	92	69.2%	349	80.6%
Yes	9	11.7%	34	15.2%	43	14.3%	6	13.3%	35	39.8%	41	30.8%	84	19.4%
Spent night outside														
No	60	77.9%	210	94.2%	270	90.0%	23	51.1%	87	98.9%	110	82.7%	380	87.8%
Yes	17	22.1%	13	5.8%	30	10.0%	22	48.9%	1	1.1%	23	17.3%	53	12.2%
Travel														
No travel	60	77.9%	210	94.2%	270	90.0%	23	51.1%	87	98.9%	110	82.7%	380	87.8%
Travel with accommodation	4	5.2%	5	2.2%	9	3.0%	18	40.0%	0	0.0%	18	13.5%	27	6.2%
Travel with outdoors stay	10	13.0%	6	2.7%	16	5.3%	0	0.0%	0	0.0%	0	0.0%	16	3.7%
Had outside activities														
No	69	89.6%	207	92.8%	276	92.0%	42	93.3%	83	94.3%	125	94.0%	401	92.6%
Yes	8	10.4%	16	7.2%	24	8.0%	3	6.7%	5	5.7%	8	6.0%	32	7.4%
Student (among those under 18 years)														
Total < 18	16	20.8%	21	9.4%	37	12.3%	12	26.7%	4	4.5%	16	12.0%	53	12.2%
No	8	50.0%	6	27.3%	14	36.8%	5	41.7%	4	100.0%	9	56.3%	23	42.6%
Yes	8	50.0%	16	72.7%	24	63.2%	7	58.3%	0	0.0%	7	43.8%	31	57.4%

Note: LLIN—Long lasting insecticidal net

In multivariate analysis (Table 4) across both districts, as the districts were combined for the analysis in order to have a sufficient sample size, those with occupations in rice agriculture, outdoor/manual work and jobs that require travel/overnight stays had higher odds of malaria infection (OR = 5.28, 95%CI (2.23, 12.60), OR = 54.90 (10.27, 293.60) and OR = 24.40 (7.60, 78.04)), respectively). Those who slept under an LLIN the previous night had a lower odds of malaria (OR = 0.44 (0.21, 0.93)) compared to those who did not sleep under LLIN. People who spent the night outside of their primary residence in the past three weeks had higher odds of malaria than those who did not (OR = 11.9 (4.99, 28.3)). Travelling whether with some accommodation (e.g., housing or a tent) or no accommodation/staying outside were associated with an increased odds of malaria (OR = 18.63 (5.52, 63.0) and OR = 5.89 (1.61, 21.6), respectively) compared to no travel. Within the occupation groups with increased odds of malaria (i.e., jobs that require travel/overnight stays and outdoor work/manual labour), itinerant vendors and miners had relatively higher odds of malaria than other occupation groups, respectively (Additional file 1: Table 1).

**Table 4:** Adjusted Odds Ratios and 95% CIs for Risk Factors of Malaria Comparing Cases and Controls

	OR (95% CI)
Primary occupation (ref- Agriculture (other than rice))	
None	2.24 (0.40, 12.6)
Rice agriculture	5.28 (2.23, 12.6)^a^
Indoor work	1.48 (0.54, 4.05)
Farm animal/fishing	2.44 (0.72, 8.22)
Outdoor work/manual labour	54.9 (10.27, 293.6)^a^
Jobs with travel/overnight stays	24.4 (7.60, 78.04)^a^
Residence in same district	0.44 (0.17, 1.18)
Sleep under LLIN	0.44 (0.21, 0.93)^a^
Night outside	11.9 (4.99, 28.3)^a^
Travel and stay (ref-no travel)	
Travel with accommodation	18.63 (5.52, 63.0)^a^
Travel with stay outside	5.89 (1.61, 21.6)^a^
Outside activities	0.95 (0.36, 2.51)

Note: Covariates adjusted include age, sex, and district (as fixed effects) and site (as a random effect)

^a^Statistically significant at alpha level of 0.05

#### Antsiranana I

In Antsiranana I, initial KIIs focused on identification of priority HRPs were conducted in March 2022 with 18 participants across 11 health catchment areas. Most participants in Antsiranana I felt that the majority of malaria cases in their health facilities or communities (e.g., those that seek care) are typically adults, followed by those aged 6–14 years and that more women than men have malaria. They also noted that the most common occupations of malaria cases were travelling vendors, fisherman, students and agriculture workers, and that high-risk activities included spending time outside their residence at night during the hot season and travel, in particular to and from Vangaindrano (south-east Madagascar) or Ambilobe (to the south in Diana Region). Travel during the harvest season was perceived as a high-risk activity. Most participants felt that people were infected with malaria outside of Antsiranana (e.g., in Vangaindrano, Analanjirofo). When asked about potential malaria cases in their community, or those that may have malaria but typically do not seek treatment at the health facility, the majority mentioned that these populations may be 21–40 years old. One participant noted that women were more inclined to go to health facilities in case of illness.

## Formative assessment

In Antsirabe II and Faratsiho, the team conducted one KII and eight FGDs—three FGDs with rice agricultural workers, one FGD with non-rice agricultural workers, and four FGDs with miners. A total of 50 people participated in these interviews and discussions. Participant miners in Antsirabe II said they often have outdoor work at night when gemstone demand is high and partake in evening social activities. Miner participants also noted the potential for malaria importation by miners traveling from higher transmission areas in Madagascar, such as Betafo, Faratsiho and Maevatanana. Rice farmer participants in both Antsirabe II and Faratsiho noted outdoor occupational activities at night during the malaria season, such as guarding the harvest in December/January and managing the field water channels. Non-rice agricultural worker participants noted that they do not work at night and always return to their primary residence for sleep. Many participants from each group reported that there was a lot of travel in June and July for “Famadihana,” which are important family celebrations.

In Antsiranana I, for the formative assessment, the team completed five FGDs with five HRP groups (farmers, students, miners, fishermen, and mobile vendors) with a total of 33 participants. Farmers and mobile vendors noted that they sleep in informal structures or work outside at night during the peak malaria season (Additional file 1: Table 2). Participants noted that students have outdoor social activities at night when mosquitoes are biting. The fishermen said they travel away from home, but only during the non-malaria season. Miners in this area reported often sleeping at their primary residences because the mines were close.

## Means of malaria prevention

In all three districts, most of the HRP participants reported not having access to LLINs. However, in Antsirabe II and Faratsiho, some rice farmers, non-rice farmers and miners reported sleeping under an LLIN, and some received IRS in their homes. All the participant HRPs reported using other means of prevention, such as burning leaves or mosquito coils, wearing long-sleeved clothing, applying oils of plants, and planting tomatoes near the house. Miners in Antsirabe II mentioned that people from Betafo cover their huts with LLINs when travelling to the district for work. Some farmers in Antsiranana I reported using old, outdated LLINs. Farmers, students, and mobile vendors in Antsiranana I reported using mosquito repellent but were not probed on how or when they used it. In this area, miners reported no use of any prevention methods, and fishermen mentioned wearing long-sleeved clothing.

## Access to healthcare

In Antsirabe II and Faratsiho, non-rice farmer participants from Faratsiho were the only participants who claimed to access the health facilities directly if they had malaria symptoms, likely due to their proximity – 7 km from the village. Most HRP participants in Antsiranana I reported having some type of access to healthcare. Rice farmers and miners in Antsirabe II and Faratsiho and fishermen and mobile vendors in Antsiranana I reported using traditional medication first and going to the health facility or CHW only if their symptoms did not resolve. Distance and cost of treatment were commonly cited barriers to healthcare access in all three districts. Some HRP participants noted that access to health facilities was worse during the rainy season. In Antsiranana I, fishermen and mobile vendor participants mentioned use of plant teas or inhalations first and reported fear of needles when being tested.

## Malaria intervention venues or times

In all three districts, participants from all of the HRP groups indicated venues (locations) where they could be accessed for a malaria intervention and provided specific times and gatekeepers, or people who have influence or control over intervention access or participation, such as community leaders. In Antsirabe II, rice farmers preferred the primary school, non-rice farmers had no preference, and miners preferred to gather at the worksites (quarries), although they were not seen as easily accessible. In Antsiranana I, all participants except farmers thought it would be possible to go through social contacts to recruit others for an intervention. In terms of locations for an intervention, fishermen and mobile vendor participants suggested holding an intervention at the port in the town of Cap Diego on any morning, while farmer participants preferred an intervention in the village centre facilitated by village headmen.

## Interest in presumptive treatment and other interventions

Most HRP group participants in the three districts, not including student participants in Antsiranana I, were interested in taking presumptive treatment, or treatment without malaria testing. In Antsirabe II and Faratsiho, miners expressed an interest in LLINs (including covering their huts with LLIN), IRS in the mines, topical repellents, and chemoprevention, and preferred community meetings or sensitization through CHWs for education campaigns. Farmers (both rice and non-rice) were interested in mass LLIN distribution, IRS, and topical repellents. Rice farmers preferred communication from health facilities, with some wanting sensitization by radio. Miners, fishermen, and mobile vendors in Antsiranana I stated a need for LLINs. Most participants preferred information from radio programs and considered village headmen and CHWs as potential gatekeepers.

## Discussion

Findings from this study show that occupations with higher odds of malaria in Antsirabe II and Faratsiho were rice agriculture, outdoor/manual work (highest risk for miners), and those with jobs that required travel or overnight stays (highest risk for itinerant vendors). In Antsiranana I, non-rice farmers, mobile vendors, and students had the highest risk of infection likely due to outdoor activities at night during the malaria transmission season. The elevated risk among these groups is tied to increased exposure due to frequent travel, staying outside overnight for work or for socializing, staying overnight in informal structures, and lack of prevention means, such as bed nets, mosquito repellents, or having house sprayed with insecticide.

Frequent travel and staying outside overnight were found to be risk factors. Participants whose occupations were identified as high risk (i.e., miners, rice farmers, other farmers, and mobile vendors) reported spending time outside at night during the malaria season, either for work, sleep, or socializing. Miner and rice farmer participants in Antsirabe II and Faratsiho reported travel to high-transmission districts such as Betafo and Maevatanana. As respondents noted, travel during the harvest season from Antsiranana I to particularly Vangaindrano, an area with higher transmission, likely leads to a higher risk of malaria in these communities. Travel and night-time outdoor activities have been shown to be major malaria risk factors in other contexts [18, 19].

Across sub-Saharan Africa, major HRP groups have included characteristics of travel and outdoor nighttime activity [18–20]. Identifying areas of high malaria transmission, tracing travel patterns and enhancing prevention and reducing transmission in these malaria hot spots is invaluable for controlling residual malaria transmission in these districts.

There is evidence that malaria transmission is often concentrated in a few population groups and is being increasingly localized among difficult-to-reach populations and those with occupations of agriculture, mining, military or forest work [21–23]. Some studies show that particularly miners [22, 24] and those with jobs that require travel have a high risk of malaria infection and low use of prevention tools [25], while studies in Nigeria and South Africa show a similar high risk of malaria infection among agricultural workers [23, 26], particularly rice-farmers [26, 27]. The risk of malaria infection is high for rice farmers because the ecological conditions of the early stages of rice fields are preferred by larvae of the vector *An. gambiae * s.l [27]. Elevated densities of *Anopheles* have also been found in rice paddy areas in Madagascar [28]. Findings from this study also showed that rice agricultural workers had 5.28 (95%CI (2.23–12.6)) times the odds of malaria compared to non-rice agricultural workers. The elevated risk of malaria in these occupation groups identified as high risk is largely reflective of occupational risk factors. Devising strategies to increase malaria protection at work sites or during work activities is key for effective malaria control.

An interesting finding in the quantitative analysis was that travel and overnight stay with accommodation was found to have a higher risk than traveling with no accommodation. However, most of the cases with travel reported a tent as their “accommodation.” The degree of protection a tent provides from risk of infection could vary by condition of the tent, use of other prevention means, and destination of the traveler. For instance, in the quantitative study, most respondents who reported sleeping in a tent went to Maevatanana, a high transmission area in Madagascar [29]. In addition, use of a tent as opposed to being outdoors could give a false sense of security and reduce use of preventive measures. Other studies show that low risk perception is likely to diminish use of preventive measures for vector-borne diseases [30], and in some places use of tents was associated with an increase in the risk of malaria [20].

This study generated information on gaps in malaria prevention and barriers to care. Across the high-risk occupations in Antsirabe II and Faratsiho, there was relatively low reported LLIN use the previous night. In the quantitative analysis, having slept under a LLIN the previous night was found to be a protective against malaria. Based on the formative assessment, in all three districts, HRP group participants used traditional medications first and reported treatment costs and distance to the health facility as barriers to access. Increasing access to ITNs and addressing treatment gaps such as cost of treatment and distance to facilities (i.e., increasing access to health care) may help reduce malaria transmission in these districts.

HRP participants in the qualitative formative assessment also provided insights on preferences for malaria treatment, prevention interventions, social and behaviour change mechanisms, and involvement of community gatekeepers. These suggestions could be trialed as subnational tailored approaches, such as presumptive treatment campaigns, which were of interest to all HRP groups, except for students. In addition to the residence-focused vector control measures of IRS and LLINs, rice farmers were interested in topical repellents, which may provide protection during the outdoor nighttime field work. Many HRP groups cited barriers to accessing malaria services from health facilities, which could be overcome through presumptive treatment or mobile screening and treatment campaigns, although costly. Integrating perspectives, preferences and solutions suggested by high-risk populations and community members, and involving gatekeepers in delivery of interventions can be useful for identifying and implementing feasible and sustainable strategies to control malaria in these districts and similar settings [31].

This study had some limitations. First, a general limitation of the case–control study approach is that populations who do not go to health facilities at all are not part of the study population. However, with more limited restrictions on health seeking, the approach was still found to be useful in identifying risk groups who may have lower access to care, primarily in areas where malaria is causing symptomatic illness. It also remains a cost-effective approach to identifying risk factors. Second, there was low sample size in Faratsiho district for the quantitative analysis, so for sufficient power, data from Antisirabe II and Faratsiho was combined in the regression. The low sample size was attributed to lower than expected malaria cases in that season, the specific reasons for which were not identified but part of typical increases and decreases in caseload. Third, the case–control study could not be extended to Antsiranana I due to projected low case numbers, so a rapid qualitative risk factor assessment was conducted instead. Fourth, the formative assessment did not achieve sample size and therefore itinerant vendors in Antsirabe II and Faratsiho were not included, and it is unknown whether saturation was reached. Fifth, as the study relied on self-reported measures, it may be subject to measurement bias, particularly recall bias. However, by restricting to the population that reported to the facilities and received testing, the test negative design used for the quantitative study is more likely to avoid differential recall of exposure and reduce confounding and selection bias that could result from differential healthcare-seeking behaviours [32]. As a result of having health officials conduct the qualitative data collection, there may have been some level of response bias, such as social desirability bias. Lastly, to reduce time and cost, the formative assessment did not collect direct quotes and instead used a rapid analytic debriefing method. As a result, there are no quotes that could have further illustrated participant perceptions.

## Conclusions

The global malaria community agrees that addressing barriers to malaria prevention and care is important for successful malaria elimination [31]. However, in subnational elimination zones, transmission is often concentrated in population groups for which service delivery is challenging because of terrain, sociodemographic factors, and mobility [6]. This study revealed more insights on Madagascar’s HRPs, their locations, potential interventions to test, and ways to access them for diagnosis, treatment, surveillance and response, prevention tools, and information that can be used for subnational tailoring of interventions.

### Supplementary Information

**Additional file 1: Table S1**. Primary occupations by district and malaria status. **Table S2.** Formative Assessment findings in the districts of Antsirabe II and Faratsiho (combined) and Antsiranana I. **Figure S1.** Monthly enrollment of cases and controls between October 2021 and March 2022 in the selected health facilities in study districts of Antsirabe II and Faratsiho.

## Data Availability

The datasets used and/or analysed during the current study are available from the corresponding author on reasonable request.

## Abbreviations

CHW: Community health worker
CI: Confidence interval
DHIS2: District Health Information System
FGD: Focus group discussion
HRP: High-risk population
KII: Key informant interview
LLIN: Long-lasting insecticide-treated net
NMCP: National Malaria Control Program
OR: Odds ratio
PMI: U.S. President’s Malaria Initiative
RDT: Rapid diagnostic test
UCSF: University of California, San Francisco

## References

[1] Shretta R, Liu J, Cotter C, Cohen J, Dolenz C, Makomva K, et al. Malaria elimination and eradication. In: Holmes KK, Bertozzi S, Bloom BR, et al. (eds). Major Infectious Diseases, 3rd Edn. Washington (DC): The International Bank for Reconstruction and Development; The World Bank. Chapter 12:2018. 30212099

[2] Killeen GF, Govella NJ, Lwetoijera DW, Okumu FO (2016). Most outdoor malaria transmission by behaviourally-resistant *Anopheles arabiensis* is mediated by mosquitoes that have previously been inside houses. Malar J.

[3] Galactionova K, Smith TA, de Savigny D, Penny MA (2017). State of inequality in malaria intervention coverage in sub-Saharan African countries. BMC Med.

[4] Ansah EK, Moucheraud C, Arogundade L, Rangel GW (2022). Rethinking integrated service delivery for malaria. PLoS Glob Public Health.

[5] Howes RE, Mioramalala SA, Ramiranirina B, Franchard T, Rakotorahalahy AJ, Bisanzio D (2016). Contemporary epidemiological overview of malaria in Madagascar: operational utility of reported routine case data for malaria control planning. Malar J.

[6] Cotter C, Sturrock HJ, Hsiang MS, Liu J, Phillips AA, Hwang J (2013). The changing epidemiology of malaria elimination: new strategies for new challenges. Lancet.

[7] Nguyen TT, Gryseels C, Tran DT, Tran DT, Smekens T, Gerrets R (2021). Understanding malaria persistence: a mixed-methods study on the effectiveness of malaria elimination strategies in South-Central Vietnam. Front Public Health.

[8] Canavati SE, Quintero CE, Haller B, Lek D, Yok S, Richards JS (2017). Maximizing research study effectiveness in malaria elimination settings: a mixed methods study to capture the experiences of field-based staff. Malar J.

[9] Smith JL, Ghimire P, Rijal KR, Maglior A, Hollis S, Andrade-Pacheco R (2019). Designing malaria surveillance strategies for mobile and migrant populations in Nepal: a mixed-methods study. Malar J.

[10] Mixed Methods Research. Harvard Catalyst: Community Engagement Program. https://catalyst.harvard.edu/community-engagement/mmr/ Accessed 10 Oct 2022.

[11] Malaria Elimination Initiative. A Malaria Elimination Guide to Targeted Surveillance and Response in High-Risk Populations. San Francisco: Institute for Global Health Sciences, University of California, San Francisco (2020). Accessed 11 Oct 2022.

[12] Reducing the impacts of climate change on key aquatic ecosystems in the central highlands, concerning the Itasy, Upper Matsiatra and Vakinankaratra regions. International Network of Basin Organizations Africa: 100 water & climate projects. Available: https://www.riob.org/en/incubation/aquatic-ecosystems-Madagascar-Central-Highlands-Madagascar 12 Oct 2022. Accessed 9 Apr 2024.

[13] Meekers D, Yukich JO (2016). The association between household bed net ownership and all-cause child mortality in Madagascar. Malar J.

[14] Madagascar Ministry of Public Health. Plan stratégique de lutte contre le paludisme Madagascar 2013–2017. (Internet). Madagascar, Antananarivo, 2013. Available from: https://endmalaria.org/sites/default/files/madagascar2013-2017.pdf

[15] Le Goff G, Léong Pock Tsy JM, Robert V (2006). Molecular characterization of the malaria vector *Anopheles*
*gambiae* s.s. in Madagascar. Med Vet Entomol.

[16] Tantely ML, Rakotoniaina JC, Tata E, Andrianaivolambo L, Fontenille D, Elissa N (2012). Modification of *Anopheles **gambiae* distribution at high altitudes in Madagascar. J Vector Ecol.

[17] Kabbale FG, Akol AM, Kaddu JB, Onapa AW (2013). Biting patterns and seasonality of *Anopheles*
*gambiae* sensu lato and *Anopheles*
*funestus* mosquitoes in Kamuli district. Uganda Parasit Vectors.

[18] Monroe A, Asamoah O, Lam Y (2015). Outdoor-sleeping and other night-time activities in northern Ghana: implications for residual transmission and malaria prevention. Malar J.

[19] Arinaitwe E, Dorsey G, Nankabirwa JI, Kigozi SO, Katureebe A, Kakande E (2019). Association between recent overnight travel and risk of malaria: a prospective cohort study at 3 sites in Uganda. Clin Infect Dis.

[20] Smith JL, Mumbengegwi D, Haindongo E, Cueto C, Roberts KW, Gosling R (2021). Malaria risk factors in northern Namibia: the importance of occupation, age and mobility in characterizing high-risk populations. PLoS ONE.

[21] Gosling RCJ, Uusiku P, Rossi S, Ntuku H, Harvard K, White C (2020). District-level approach for tailoring and targeting interventions: a new path for malaria control and elimination. Malar J.

[22] Jacobson JO, Cueto C, Smith JL, Hwang J, Gosling R, Bennett A (2017). Surveillance and response for high-risk populations: what can malaria elimination programmes learn from the experience of HIV?. Malar J.

[23] Naidoo S, London L, Burdorf A, Naidoo RN, Kromhout H (2011). Occupational activities associated with a reported history of malaria among women working in small-scale agriculture in South Africa. Am J Trop Med Hyg.

[24] Olapeju B, Adams C, Hunter G (2020). Malaria prevention and care seeking among gold miners in Guyana. PLoS ONE.

[25] Weber R, Schlagenhauf P, Amsler L, Steffen R (2003). Knowledge, attitudes and practices of business travelers regarding malaria risk and prevention. J Travel Med.

[26] Babamale OA, Opeyemi OA, Bukky AA, Musleem AI, Kelani EO, Okhian BJ (2020). Association between farming activities and *Plasmodium falciparum* transmission in rural communities in Nigeria. Malays J Med Sci.

[27] Chan K, Tusting LS, Bottomley C, Saito K, Djouaka R, Lines J (2022). Malaria transmission and prevalence in rice-growing versus non-rice-growing villages in Africa: a systematic review and meta-analysis. Lancet Planet Health.

[28] Arisco NJ, Rice BL, Tantely LM, Girod R, Emile GN, Randriamady HK (2020). Variation in *Anopheles* distribution and predictors of malaria infection risk across regions of Madagascar. Malar J.

[29] Ménard D, Ratsimbasoa A, Randrianarivelojosia M, Rabarijaona L-P, Raharimalala L, Domarle O (2008). Assessment of the efficacy of antimalarial drugs recommended by the National Malaria Control Programme in Madagascar: up-dated baseline data from randomized and multi-site clinical trials. Malar J.

[30] Aerts C, Revilla M, Duval L, Paaijmans K, Chandrabose J, Cox H (2020). Understanding the role of disease knowledge and risk perception in shaping preventive behavior for selected vector-borne diseases in Guyana. PLoS Negl Trop Dis.

[31] Dhiman S (2019). Are malaria elimination efforts on right track? An analysis of gains achieved and challenges ahead. Infect Dis Poverty.

[32] Sullivan SG, TchetgenTchetgen EJ, Cowling BJ (2016). Theoretical basis of the test-negative study design for assessment of influenza vaccine effectiveness. Am J Epidemiol.

