# Supplementary material for: Identifying and characterizing high-risk populations in pilot malaria elimination districts in Madagascar: a mixed-methods study
Source: Malar J. 2024 Apr 26;23:121. doi: 10.1186/s12936-024-04927-w (PMC11046788; doi:10.1186/s12936-024-04927-w)
Supplement: Supplementary file 1 — Additional file 1: Table S1. Primary occupations by district and malaria status. Table S2. Formative Assessment findings in the districts of Antsirabe II and Faratsiho (combined) and Antsiranana I. Figure S1. Monthly enrollment of cases and controls between October 2021 and March 2022 in the selected health facilities in study districts of Antsirabe II and Faratsiho. [file 12936_2024_4927_MOESM1_ESM.docx]

**Additional file 1: Table S1**. Primary occupations by district and malaria status

|  | **Antsirabe II** | | | | **Faratsiho** | | | | **Total** | | | |  |  |
| --- | --- | --- | --- | --- | --- | --- | --- | --- | --- | --- | --- | --- | --- | --- |
| **Primary Occupation** | **case** | | **control** | | **case** | | **control** | | **case** | | **control** | | **Grand total** | |
| Agriculture (other than rice) | 23 | 29.9% | 146 | 65.5% | 1 | 2.2% | 25 | 28.4% | 24 | 19.7% | 167 | 53.7% | 191 | 44.1% |
| Construction worker | 2 | 2.6% | 0 | 0.0% | 0 | 0.0% | 0 | 0.0% | 2 | 1.6% | 0 | 0.0% | 2 | 0.5% |
| Elevage (animal husbandry) | 7 | 9.1% | 12 | 5.4% | 0 | 0.0% | 5 | 5.7% | 7 | 5.7% | 17 | 5.5% | 24 | 5.5% |
| Itinerant vendor | 16 | 20.8% | 4 | 1.8% | 6 | 13.3% | 4 | 4.5% | 22 | 18.0% | 8 | 2.6% | 30 | 6.9% |
| Miner | 4 | 5.2% | 2 | 0.9% | 4 | 8.9% | 0 | 0.0% | 8 | 6.6% | 2 | 0.6% | 10 | 2.3% |
| None | 0 | 0.0% | 4 | 1.8% | 2 | 4.4% | 2 | 2.3% | 2 | 1.6% | 10 | 3.2% | 12 | 2.8% |
| Nurse, Teacher, Professional | 1 | 1.3% | 7 | 3.1% | 0 | 0.0% | 2 | 2.3% | 1 | 0.8% | 9 | 2.9% | 10 | 2.3% |
| Other | 1 | 1.3% | 0 | 0.0% | 0 | 0.0% | 0 | 0.0% | 1 | 0.8% | 0 | 0.0% | 1 | 0.2% |
| Producer of flour | 0 | 0.0% | 1 | 0.4% | 0 | 0.0% | 0 | 0.0% | 0 | 0.0% | 1 | 0.3% | 1 | 0.2% |
| Rice agriculture | 15 | 19.5% | 29 | 13.0% | 31 | 68.9% | 46 | 52.3% | 46 | 37.7% | 75 | 24.1% | 121 | 27.9% |
| Rock cutter, quarry worker | 1 | 1.3% | 0 | 0.0% | 1 | 2.2% | 0 | 0.0% | 2 | 1.6% | 0 | 0.0% | 2 | 0.5% |
| Sales or commerce – small market (vendor) | 2 | 2.6% | 6 | 2.7% | 0 | 0.0% | 2 | 2.3% | 2 | 1.6% | 8 | 2.6% | 10 | 2.3% |
| Student | 5 | 6.5% | 12 | 5.4% | 0 | 0.0% | 2 | 2.3% | 5 | 4.1% | 14 | 4.5% | 19 | 4.4% |
| Total | 77 |  | 223 |  | 45 |  | 88 |  | 122 |  | 311 |  | 433 |  |

**Additional file 1: Table S2.** Formative Assessment findings in the districts of Antsirabe II and Faratsiho (combined) and Antsiranana I

|  | **Antsirabe II and Faratsiho (N=50)** | **Antsiranana I (N=33)** |
| --- | --- | --- |
| **Groups selected for KII & FGD** | Rice agricultural workers, non-rice agricultural workers, and miners. | Farmers, students, miners, fishermen, and mobile vendors. |
| **1. Occupations with highest risk** | *Miners* | *Farmers and mobile vendors* |
|  | Reasons include working overnight, evening socializing and travel to high-transmission districts Betafo and Maevatanana. | Reasons include sleeping in informal structures and working at night during the peak malaria season. |
|  | *Rice farmers* | *Students* |
|  | Reasons include guarding harvest in malaria season (Dec/Jan), working overnight when managing water channels and travel for “Famadiana,” or exhumations. | Reasons include outdoor social activities at night. |
| **2. Means of prevention** | Most do not have LLINs. | No access to LLINs. |
|  | Other means of prevention including burning leaves or mosquito spirals, wearing long-sleeved clothing, applying oils of plants, and planting tomatoes near the house. | Farmers, students, and mobile vendors have used mosquito repellent.  Fishermen have worn long-sleeved clothing. |
| **3. Access to healthcare** | Only non-rice farmers from Faratsiho reported access to health facilities. Rice farmers and miners have used traditional medication first. | Most had some type of access to health care. Fishermen and mobile vendors used traditional plant remedies first. |
|  | Barriers include distance to health facilities and treatment cost. | Barriers include distance, medication cost, and fear of needles. |
| **4. Malaria intervention venues or times** | Rice farmers preferred the primary school or college on Fridays or Saturdays, either with CHW or village leaders.  Non-rice farmers have no preferences except for advance notice. | All except farmers thought it would be possible to go through social contacts to recruit others for an intervention. Fishermen and mobile and mobile vendors suggested Port Cap Diego while farmers preferred Mondays in the village center. |
|  | Feasible intervention for miners would be at the village or, if close by, at the mining sites. | Farmers preferred intervention during mornings in the village center. |
|  |  | Fishermen and mobile vendors suggested mornings at the Port Cap Diego. |
| **5. Interest in presumptive treatment and other interventions** | Most were interested in presumptive treatment.  Miners stated an interest in LLINs, IRS in the mines, topical repellents, chemoprevention, and the idea to cover their huts with LLINs, similar to the miners originating from Betafo. Farmers (both rice and non-rice) were interested in mass LLIN distribution, IRS, and topical repellents.  Miners preferred community meetings or through CHWs while rice farmers preferred communication from health facilities with some wanting sensitization by radio. | All groups except students were interested in presumptive treatment. Miners, fishermen, and mobile vendors stated a need for LLINs.  Students asked for more information on reproductive health.  Most groups preferred information from radio programs and gatekeepers were considered village headmen, health facilities, and CHWs. |

**Additional file 1: Figure S1.** Monthly enrollment of cases and controls between October 2021 and March 2022 in the selected health facilities in study districts of Antsirabe II and Faratsiho
